# Periodontal Disease and Pregnancy: Correlation with Underweight Birth

**DOI:** 10.1055/s-0042-1757906

**Published:** 2022-12-13

**Authors:** Giuseppe Minervini, Manuele Basili, Rocco Franco, Patrizio Bollero, Maura Mancini, Lucia Gozzo, Giovanni Luca Romano, Maria Maddalena Marrapodi, Francesca Gorassini, Cesare D'Amico, Eugenio Pedullà, Luca Fiorillo

**Affiliations:** 1 Multidisciplinary Department of Medical-Surgical and Odontostomatological Specialties, University of Campania Luigi Vanvitelli, Naples, Italy; 2 Department of Biomedicine and Prevention, University of Rome “Tor Vergata,” Rome, Italy; 3 Department of Systems Medicine, University of Rome “Tor Vergata,” Rome, Italy; 4 Unit of Ophthalmology, Department of Scienze Biomediche, Odontoiatriche e Delle Immagini Morfologiche e Funzionali, University of Messina, Messina, Italy; 5 Clinical Pharmacology Unit, Regional Pharmacovigilance Centre, University Hospital of Catania, Catania, Italy; 6 Department of Biomedical and Biotechnological Sciences, University of Catania, Catania, Italy; 7 Department of Woman, Child and General and Specialist Surgery, University of Campania “Luigi Vanvitelli,” Naples, Italy; 8 Department of Biomedical and Dental Sciences, Morphological and Functional Images, University of Messina, G. Martino Polyclinic, Messina, Italy; 9 Department of General Surgery and Surgical-Medical Specialties, School of Dentistry, University of Catania, Catania, Italy; 10 Department of Prosthodontics, Dr. D.Y. Patil Dental College and Hospital, Dr. D.Y. Patil Vidyapeeth, Pimpri, Pune, India

**Keywords:** oral health, pregnancy, periodontal disease, underweight birth, periodontal disease

## Abstract

Periodontal disease is a risk factor for many systemic diseases including preterm birth and underweight birth. The purpose of this systematic review is to analyze the literature and to highlight any clinical correlation. Information sources such as PubMed, MEDLINE, and Web of Science were consulted to obtain our results with these keywords “periodontal disease,” “pregnancy,” “weight loss” using the connector “AND.” After the first screening by authors, only 27 articles were included in this review. From the analysis of the literature, it was noted that the presence of periodontal disease could have a correlation with underweight birth. Surely, control oral hygiene and oral health is essential during pregnancy to reduce risks, and these results should be essential in establishing a protocol to be maintained during pregnancy.

## Introduction

Periodontal disease is a chronic inflammation of the oral cavity, caused by bacteria, that stimulates an inflammatory response and causes the destruction of the supporting tissues of the tooth. 1 2 3 4 5 The clinical manifestations of periodontal disease range from periodontitis to gingivitis, causing the destruction of the periodontium and thus the loss of the tooth. 6 7 Activation of the inflammatory response is due to the release of metalloproteinases and prostaglandins which stimulate the activation of osteoclasts and therefore bone resorption. All of these inflammatory mediators can spread through the bloodstream and spread throughout the body. 6 Hormonal changes in pregnancy, especially estrogen and progesterone, are an aggravating factor in periodontal disease. 8 When there is lack of hygiene, this pathology can affect up to 60% of women. 9 Periodontal disease during pregnancy can trigger a state of chronic inflammation spread throughout the body. 10 11 12 Several studies have correlated periodontal disease and problems during pregnancy and the newborn. Some authors correlated periodontal disease with increased risk of preterm birth. Some complications have been related in pregnant women with periodontal disease including gestational diabetes, preeclampsia, intrauterine growth restriction, early abortion, premature birth, low birth weight (LBW), and a higher risk of early neonatal infection. 13 However, these associations have not yet been ascertained. 14 15 The first to hypothesize a possible association between periodontal disease and childbirth was Offenbacher in 1996. He hypothesized a possible association between periodontal disease and adverse events during childbirth including preterm, LBW, early miscarriage, gestational diabetes, and preeclampsia. 16 Periodontal disease affects several biological pathways, increasing the levels, not only in crevicular fluid 17 but also in plasma, of interleukin (IL)-1 beta, IL-6, tumor necrosis factor alpha (TNF-α), beta-glucuronidase (β-glucuronidase), prostaglandin E2 (PGE2), aspartate aminotransferase (AST), metalloproteinase-8 (MMPT-8), and decreased osteoprotegerin (OPG) level. 11 18 19 All of these mediators are able to penetrate the chorioamniotic barrier. Various microbes are able to penetrate this barrier and therefore come into contact with the fetus. All these inflammatory products cause the local release of PGE2 and TNF-α which cause the rupture of the membrane and consequently the preterm birth. This environment could cause a decrease in fetal growth. 20 Few interventions in the mouth are possible during pregnancy. The treatments that can be performed for the treatment of periodontal disease are limited. Acting during pregnancy to treat periodontal disease may not be effective in reducing systemic inflammation. Root treatment can cause bacteremia, leading to serious consequences. 21 Furthermore, *Porphyromonas gingivalis* is the most studied periodontal pathogen. This pathogen, together with *Prevotella intermedia* and *Treponema denticola* , is very common in pregnant women. *Porphyromonas gingivalis* creates an alteration of the oral microbiota. The mechanisms by which *P. gingivalis* acts are not fully understood, due to the lack of an efficient animal model. The action of *P. gingivalis* was performed on a mouse model on which ligatures were made around the teeth to allow for an accumulation of plaque. 22 23 Mouse model experiments were performed during pregnancy. In order to evaluate the role of *P. gingivalis* , experiments were performed in a mouse model with or without *P. gingivalis* infection. Increased inflammation was noted in ligated mice without *P. gingivalis* colonization; however, increased inflammatory status was noted in mice with *P. gingivalis* . By comparing mice infected with *P. gingivalis* infection and those without *P. gingivalis* infection, we illustrated the role of *P. gingivalis* in promoting disease. 24 The study evaluated the articles in the literature concerning the correlation between birth weight and periodontal disease. Therefore, our interest has turned to evaluating all the literature to verify a correlation between periodontal disease in the mother and underweight birth. Afterward, we evaluate the effects of periodontal therapy on birth weight.

## Methods

The study was conducted utilizing the main scientific databases (PubMed, MEDLINE, and Web of Science). The time window considered for the electronic search was from 01/03/2020 to 01/03/2021. The term “periodontal disease” was combined with “pregnancy” and with “weight loss” using the connector “AND.” The web search was assisted by the use of Medical Subjects Headings. The criteria for this review are described in the Preferred Reporting Items for Systematic reviews and Meta-Analyses (PRISMA) and by the following flow diagram. The purpose of this review was to answer the following question using a PICO method (P: patient problem/population; I: intervention; C: comparison; O: outcome): Is periodontal disease a predisposing factor for underweight birth?

The following inclusion criteria were used: articles in English, human studies, clinical trials. The following exclusion criteria were used: articles which did not answer the key questions, case reports, duplicate articles, books, letters to editors, and experimental studies.

The systematic review was recorded in the International PROSPERO (International Prospective Register of Systematic Reviews) database with the following number CRD42022327426 on April 21, 2022.

### Risk of Bias (According to PRISMA 2020)

**Fig. 1:**
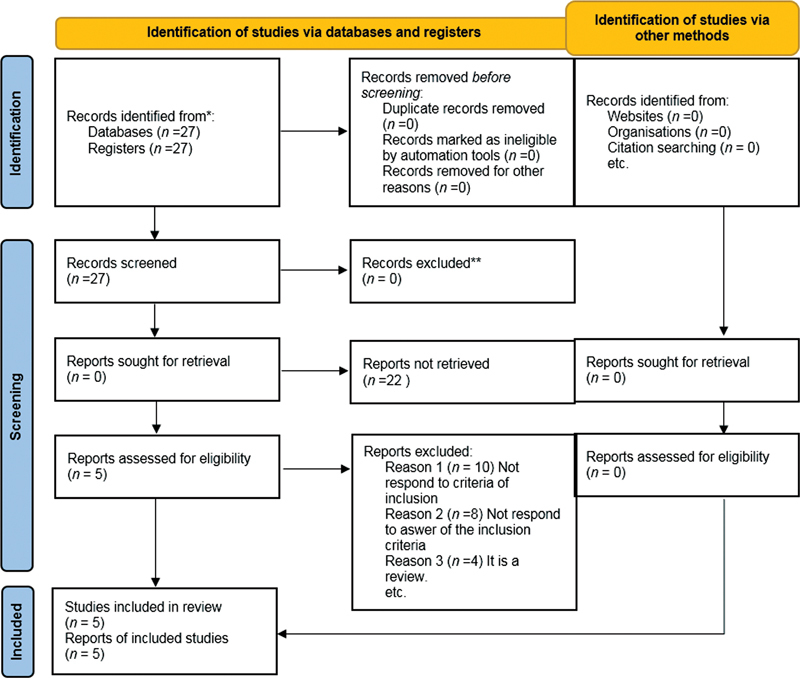
Preferred Reporting Items for Systematic reviews and Meta-Analyses flowchart.

The risk of bias was solved in this way: the articles were collected and read by two independent authors, the same authors read and included or excluded the articles independently, and a lack of agreement was resolved by a third author. All authors used the same terms and the same inclusion and exclusion criteria ( Fig. 1 ) ( Table 1 ).

**Table 1:** Risk of bias definition

	Jiang et al a	Gomes-Filho et alb	López et al a	Sadatmansouri et al a	Michalowicz et al a
Random sequence generation	Low	High	Low	Low	Low
Allocation concealment	Low	Low	Low	Low	Low
Blinding of outcome	Low	Low	Low	Low	Low
Selective reporting	Low	Low	Low	Low	Low
Other bias	Low	Low	Low	Low	Low

a Blinded study and an optimal protocol of inclusion and exclusion criteria.

b All patients had a low socioeconomic origin.

## Results

In the research phase they were selected, 27 articles were selected after entering the keywords in the database. During the screening phase, the abstracts were read and the articles were seen to meet the inclusion and exclusion criteria. After reading, 22 articles were excluded as they did not meet the established criteria and did not answer the questions of the PICO.

In the final phase, five articles were selected and analyzed. Jiang et al analyzed 470 female patients with the intention of conceiving within 1 year and suffering from periodontal disease were recruited. The patients were divided into two groups. The study group received periodontal therapy, scaling and root planing, and oral hygiene education. 25 26 The control group will only receive supragingival prophylaxis and oral hygiene education. The women were monitored throughout pregnancy. Periodontal indices during pregnancy and the weight of the infant and the duration of gestation were evaluated. Periodontal indices were calculated at 32 and 36 weeks and probing depth, loss of clinical attachment, and bleeding rate on probing were evaluated.

The study showed how an improvement in oral hygiene led to a benefit and a higher birth weight of the newborn. 21 The Gomes-Filho et al's study analyzed the association between periodontal disease and low weight at birth through an experimental study. A total of 234 women were recruited. The sample consisted of 234 pregnant women: 54 women were assigned to the study group in which the treatment of periodontal disease was carried out; 68 nonperiodontal disease women were assigned to control group I, while in the control group II, made up of women with untreated periodontal disease, 112 women were assigned. All patients underwent a thorough periodontal examination, all indices of periodontal disease such as probing depth, gingival recession, loss of clinical attachment, and bleeding index were evaluated. The women in the study group received random periodic therapy and supportive therapy throughout pregnancy. After birth, the periodontal indices were recalculated and the weight of the newborn was measured at birth in all three groups. The variables were measured using the chi-square and Fisher's tests. The significance level was set at 5%. It was obtained, thanks to the statistical analysis, that the birth weight was similar in the study group and the control nonperiodontopathic woman group. The findings suggest that periodontal therapy helps and enables infants to have a normal birth weight. 11 27 López et al's study evaluated the association between periodontal disease and LBW risk. A randomized controlled study was performed; 400 women between the ages of 18 and 35 years undergoing antenatal care in Chile were enrolled. At random, 200 women were assigned to the study group ( *n* = 200) and the others were assigned to the control group ( *n* = 200). The study group received periodontal treatment before 28 weeks, while the control group received periodontal treatment after delivery. The calculated values were the calving at less than 37 weeks and the weight less than 2,500 g. Forty-nine women were excluded from the study for various reasons. LBW has an incidence of 1.84% (3/163) in the study group and 10.11% (19/188) in the control group (odds ratio [OR]: 5.49, 95% confidence interval [CI]: 1.65–18.22, *p* = 0.001). Statistical analysis evaluated by multivariate logistic regression found that periodontal disease was the factor most strongly associated with LBW (OR: 4.70, 95% CI: 1.29–17.13). Therefore, periodontal disease has association with LBW and therefore periodontal therapy significantly reduces birth weight rates. 28 Sadatmansouri et al's study evaluated the incidence of periodontal disease, preterm delivery, and LBW. The aim of this study was to evaluate the effects of periodontal therapy on birth weight. The study was carried out on 30 women between the ages of 18 and 35 years who suffer from periodontitis. Fifteen women underwent periodontal therapy including scaling and root planing, together with the use of 0.2% mouthwash for 1 week. No therapy was performed in the control group. After various checks, statistical analysis was carried out for the assessment of birth weight and preterm birth. This study showed that in the control group, LBW was present in 26.7%, while in the study group, there were no infants with this condition ( *p* < 0.05). The mean weight of the infants in the study group was 3,059.3-389.7 g in the study group and 3,371-394.2 g in the control group ( *p* < 0.05). Periodontal therapy, phase I, results in a reduction in the incidence rate of preterm LBW. Therefore, the application of such a simple method is recommended among pregnant women with periodontal diseases. 29 Michalowicz et al's study evaluated the possible association between periodontitis and adverse events during the gestation phase. Birth data collected during childbirth with periodontal studies were used and combined. The women were randomly selected and 413 received periodontal treatment before 21 weeks of gestation and 410 women after delivery. Periodontitis refers to a loss of clinical attachment of less than 3 mm. The data were compared using a *t* -test between treated and untreated women. The results showed that the mean birth weight (3,295 vs. 3,184 g, *p* = 0.11) had no statistically significant differences between the two groups. Age and weight are not associated with the change in periodontal indices ( *p* > 0.05). Worsening of periodontal disease is not associated with LBW or preterm birth 30 ( Table 2 ).

**Table 2:** Summary of the analyzed data

Articles	Samples	Results	Clinical significance
Jiang et al	470 females divided into study and control groups	The study showed how an improvement in oral hygiene led to a benefit and a higher birth weight of the newborn	Improved periodontal health affects birth weight
Gomes-Filho et al	A sample of 234 women; 54 in the study group, 54 in the control group 1 and 112 in the control group 2	Study group received periodontal therapy and control group random and periodic control. The birth weight was higher in the study group	Improved periodontal health affects birth weight
López et al	400 women divided in the study group and control group	Study group received periodontal therapy and control group placebo. The birth weight was higher in the woman with periodontal therapy	Improved periodontal health affects birth weight
Sadatmansouri et al	40 women divided in the study group and control group	Study group received periodontal therapy and control group placebo. Lower birth weight have a high incidence in control group	Improved periodontal health affects birth weight
Michalowicz et al	A sample of 823 women divided in 413 study group and 410 control group	Study group received periodontal therapy and control group placebo. No statistical significance in two groups of birth weight	No statistical signifiance

## Discussion

Recent research has shown the association between some systemic conditions such as atherosclerosis, myocardial infarction, stroke, and premature birth. The studies analyzed, most of them, have amply shown how periodontal pathologies are a favoring factor in the birth of premature and underweight infants. While there may be other contributing factors, its importance should not be underestimated. 31 The pathogenetic mechanism is still being defined; however, the mechanisms attributed to preterm birth are the translocation of periodontal bacteria or bacterial products including lipopolysaccharide into the placenta. All of this causes the release of biochemical mediators that result in preterm birth. Another factor that causes preterm delivery is the pathogenetic mechanisms that have been hypothesized to cause low preterm birth weight as a consequence of periodontal infection are the migration of endogenous cytokines including TNF-α, IL-1, IL-6, and PGE2 from the periodontium directly into the placenta. 32 Animal model studies have shown that periodontal infection, specifically *P. gingivalis* , retarded fetal growth. Preliminary studies conducted at the University of North Carolina further confirmed these conclusions. Mothers with periodontal disease showed a seven times higher risk of preterm birth compared with periodontally healthy mothers. 14 In another study, the levels of PGE2 and periodontal pathogens were evaluated and their values were higher in mothers with preterm birth and with periodontal disease. LBW is a major socioeconomic health problem worldwide. These are problems that lead to health implications, as well as problems at the child's level such as neurodevelopmental abnormalities and developmental abnormalities. 13 Regarding oral health, LBW and premature birth cause problems in calcification of the teeth due to an alteration of the correct balance of calcium. In fact, there is hypoplasia of the deciduous teeth. 13 33 34 Intubation of the newborn causes a groove at the palatal level, creating an alteration in the level of growth. There is also a late eruption of the dentition. 35 36 Therefore, prevention in pregnant women is of particular importance. 37

These analyzed studies showed that periodontal attachment loss was significantly higher in underweight and preterm infants. The study by Offenbacher et al (1996) confirmed this hypothesis that the severity of periodontitis is higher in the study group than the control group. 38 On the contrary, a study by Michalowicz et al 30 did not show this statistical correlation. Studies by Offenbacher et al 38 and Mitchell-Lewis et al 39 did not show an association between bleeding index and low preterm birth weight. Pocket probing depth also had a statistically significant correlation in women who had a preterm pregnancy or LBW. In the present study, the Oral Hygiene Index-Simplified score was statistically significant compared with a study by Dasanayake et al, which showed that the mothers of the cases had more sextants with the calculation. 32 Furthermore, some studies (Offenbacher et al [1998]) have shown that the female gender is a factor favoring LBW. Certainly, a control and management of periodontal disease is certainly a factor favoring underweight birth. The dentist must therefore help the future mother to prevent the development of periodontal disease. The study assessed the need for an improvement in the conditions of oral hygiene in pregnant women in order to allow for a healthy maturation and birth of the infant. It is now well established that the bacteria responsible for periodontal disease are responsible for many systemic diseases; therefore, this trend has also opened this hypothesis. Some bacteria responsible for periodontal disease can migrate into the fetus and pass the placenta and can interfere with the growth of the fetus; moreover, the systemic inflammatory state of the mother interferes, due to the increase in prostaglandins, blocks, or slows the growth of the fetus. Therefore, all dentists must pay attention in the preventive phase and try to cure or intercept periodontal pathology in pregnant women as well as a reason for primary prevention in the mother also in the unborn child. The guidelines provide periodic checks during pregnancy every 3 months and periodontal maintenance therapies throughout the gestation phase. However, further studies are needed to confirm this hypothesis; however, all the studies taken into consideration have shown this correlation.
